# First Reported Case of Femoral Facial Syndrome in an Adult: Esophageal Adenocarcinoma as a Progressive Gastrointestinal Manifestation

**DOI:** 10.7759/cureus.24285

**Published:** 2022-04-19

**Authors:** Cynthia A Reyes, Juliana N Young, Paul R Torres

**Affiliations:** 1 Pediatrics, Burrell College of Osteopathic Medicine, Las Cruces, USA; 2 Medicine, Burrell College of Osteopathic Medicine, Las Cruces, USA; 3 General Surgery, Lovelace Regional Hospital, Roswell, USA

**Keywords:** barrett's esophagus (be), esophagogastroduodenoscopy (egd), gastroesophageal reflux disease (gerd), esophageal stricture, esophageal adenocarcinoma, femoral hypoplasia with unusual facies syndrome (fhufs), femoral facial syndrome (ffs)

## Abstract

A 42-year-old female with a past medical history of femoral facial syndrome (FFS) and years of gastroesophageal reflux disease presented to our clinic with symptoms of dysphagia and iron deficiency anemia. On upper endoscopy, esophageal stricture and adenocarcinoma were detected. Unfortunately, the patient developed coronavirus disease 2019 (COVID-19) multi-organ failure prior to cancer treatment and died with dignity after choosing comfort care measures. To the best of our knowledge, we report the first case of FFS in an adult patient. This case also uniquely highlights the rare gastrointestinal manifestations of FFS.

## Introduction

Femoral hypoplasia with unusual facies syndrome (FHUFS), now known as femoral facial syndrome (FFS), is a rare condition categorized by femoral hypoplasia with the presence of at least two facial anomalies such as micrognathia, cleft palate, thin upper lip, short nose, up-slanting palpebral fissures, and low set ears [1-5]. These criteria are essential to help differentiate FFS from other similarly presenting conditions such as caudal dysplasia syndrome [1,2,6-8]. Literature on FFS is limited with approximately 70 reported cases worldwide, affecting African American, Arab, Caucasian, and Chinese populations as of 2021 [1,3,4,6,9]. Research has focused on orthopedic management and detection of FFS in utero and cases have mainly been documented in neonates, infants, and, rarely, toddlers [3-7,10,11]. Life expectancy in FFS is currently unknown. Phenotypic expression of FFS most commonly includes musculoskeletal, craniofacial, and spinal malformations [5]. Genitourinary, cardiac, and central nervous system defects have also been recognized [3]. Gastrointestinal system manifestations are rare in FFS with three separate anomalies reported to date [12,13]. Currently, there are no guidelines for management given the incredible rarity of this condition [3].

This case presents the following gastrointestinal manifestations in the first adult patient recognized with FFS: long-standing gastroesophageal reflux disease (GERD), shortened esophagus, esophageal stricture, Barrett’s esophagus, and esophageal adenocarcinoma. To the best of our knowledge, this is the first reported case of FFS in an adult. This case report elucidates the previously unknown possibility that those affected by FFS may have decades-long lifespans and brings attention to the potential development of adenocarcinoma that may be exacerbated by skeletal and gastrointestinal manifestations in individuals with FFS who survive outside of the womb. 

## Case presentation

A 42-year-old female with FFS was referred to our general surgery gastrointestinal clinic complaining of dysphagia, food regurgitation, and low energy. She initially presented to the emergency room with complaints of heartburn, chronic reflux, and two months of progressively worsening dysphagia. Marked iron deficiency anemia was present at this time with hemoglobin levels less than 6gm/dL. However, despite multiple attempts, an intravenous line could not be started and therefore was unable to be transfused with blood. She was placed on iron supplements. Four months later, she returned to the emergency room with dysphagia. It is important to note that around this time her father had passed away. In an effort to evaluate a possible underlying oropharyngeal cause of her symptoms, a CT soft tissue neck without contrast was ordered, which revealed oral contrast in the proximal esophagus but no soft tissue abnormalities. Referral for esophagogastroduodenoscopy (EGD) was made, and the patient presented to our clinic two weeks later.

**Figure 1:**
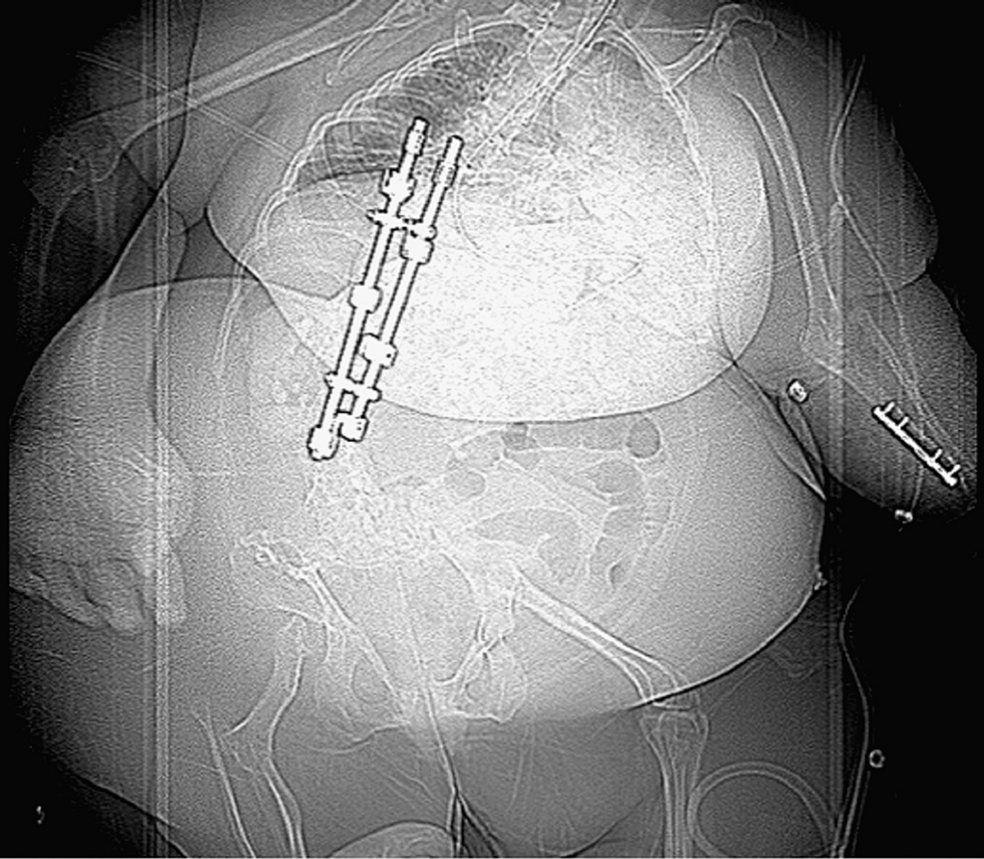
Femoral Facial Syndrome in a 42-year-old Female Asymmetric femoral hypoplasia with completely absent right femur and dislocated right fibula and tibia. Dextroscoliosis of thoracolumbar spine with posterior fusion fixation metal hardware. In addition, bilateral shortened upper extremities are evident with dislocated left radial head and left ulna open reduction internal fixation hardware.

**Figure 2:**
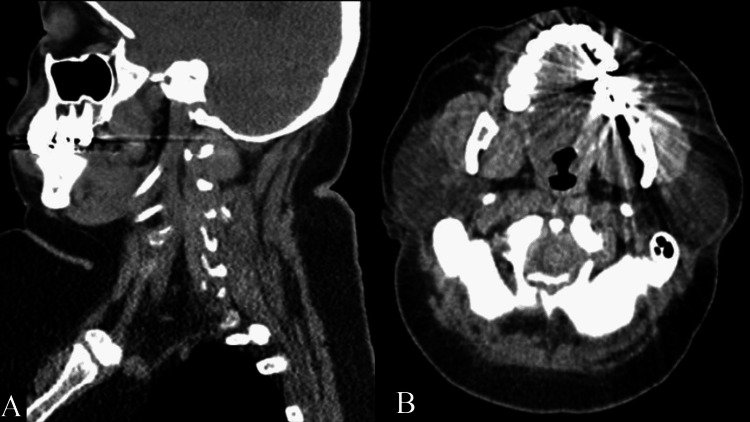
CT Head Without Contrast: Facial Anomalies Evident in Femoral Facial Syndrome (A) Sagittal view; illustrating micrognathia, very small mouth, and short nose. In addition, shortened neck and esophagus are also appreciated. (B) Axial view; surgically corrected cleft lip/palate.

On presentation at our clinic, the patient was observed to be an obese adult female of shorter than average height measuring 2’ 5” and exhibited classic FFS manifestations. The patient was wheelchair-bound and had asymmetric femoral hypoplasia with a completely absent right femur and shortened left femur. In addition, she had a dislocated right fibula and tibia, unequal shortened upper extremities with dislocated left radial head and left ulna open reduction internal fixation hardware, dextroscoliosis with rod spinal stabilization, posterior fusion thoracolumbar spinal fixation, and bilateral talipes equinovarus with toe overlapping (Figure 1). The patient’s distinctive facial features included a short broad-tipped nose, long philtrum, thin upper lip, and very small mouth. Micrognathia, low set posteriorly rotated ears, surgically corrected cleft lip/palate, and a short neck were also present (Figure 2).

The patient presented with complaints of progressively worsening dysphagia over six months and could no longer tolerate solid foods, eating a thick liquid diet in small volumes, which also proved difficult for the patient to swallow. Difficulty was also present in swallowing liquids; however, hydration and voiding were still intact. The patient reported the sensation of food getting stuck in her throat and regurgitated food at least once daily. In addition, the patient had a history of frequent heartburn and chronic GERD for the past several years. There was minimal relief with Tums, and the patient had taken esomeprazole 20mg daily for the past year and a half. At the time of presentation to our clinic, lab results indicated iron deficiency hypochromic microcytic anemia with thrombocytosis. Hemoglobin levels were at a sufficient level to proceed with the EGD.

**Figure 3:**
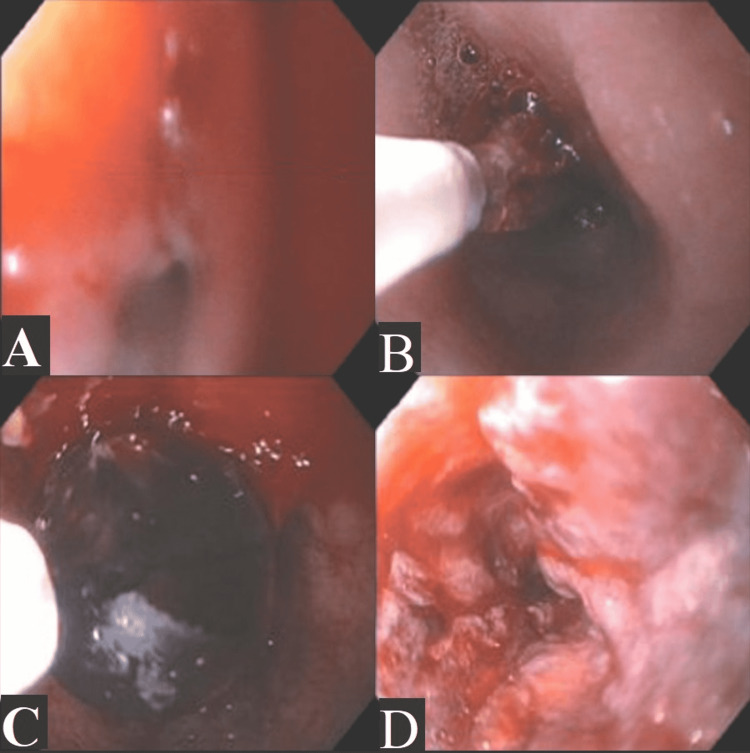
Esophagogastroduodenoscopy (A) Esophageal stricture (4mm x 5cm) located in middle third of the esophagus at 20cm. (B) Esophageal stricture being stretched during balloon dilation to allow further investigation of her upper gastrointestinal tract. (C) Esophageal stricture dilated to 13.5mm after balloon dilation. (D) Invasive esophageal adenocarcioma located in lower third of the esophagus at 25cm.

A week later, the patient underwent EGD for which proper anesthetic was essential. The bilateral glossopharyngeal nerve block was performed prior to insertion of a pediatric-sized bite guard and tube, which revealed a shortened esophagus less than 30cm from incisors to esophageal-gastric junction and 3cm hiatal hernia. One severe proximal esophageal stricture measuring 4mm x 5cm was present (Figure 3A), requiring balloon dilation up to 13.5mm to continue the examination of the upper gastrointestinal tract (Figure 3B, Figure 3C). The distal esophagus (25cm) displayed intestinal metaplasia consistent with Barrett’s esophagus. Biopsy at the distal esophagus (25cm) later revealed invasive esophageal adenocarcinoma with tubular, mucinous, and signet ring features (Figure 3D).

In addition, gastric biopsy disclosed chronic mild gastritis without the presence of *Helicobacter pylori.* Unfortunately, days later, the patient presented to the emergency room and was found to be positive for COVID-19. The patient quickly deteriorated, going into septic shock, renal failure, and acute respiratory failure. Less than a week after the EGD procedure, the patient died as a result of COVID-19 complications, despite being vaccinated. The patient was therefore never referred to a tertiary care center for adequate cancer staging and management. Given her poor prognosis, the patient and her husband ultimately chose to pursue comfort care measures. The patient died spending the last moments of her life with dignity: pain-free and supported by her loved ones.

## Discussion

In this report, we document the first case of an adult with FFS and discuss the profound impact of this condition on the gastrointestinal system. Cases in the literature have reported the earliest diagnosis of FFS in utero at 12-15 weeks’ gestation, with the latest previously reported viability up to three years of age [3-7,10,11].

The manifestations of FFS and the definitive cause of mortality have never been fully discerned. Although some cases describe the cause of death to be termination of the pregnancy [4,13], others describe that death can occur shortly prior to and after birth [9]. It is possible that early fetal and infant deaths were complications caused by maternal diabetes, oligohydramnios, or an unknown risk factor associated with FFS [3,5,12]. It is unknown if there have been reported deaths in toddlers who have FFS as case reports primarily describe orthopedic and psychosocial complications [1,3]. As this is the first known reported case of FFS in an adult, the cause of mortality in those that live past infancy is unknown. This case report therefore further suggests people with FFS may live longer, relatively healthy lives than previously recognized. 

Features of FFS can be detected via prenatal ultrasound in the late first or beginning of the second trimester [4]. Although FFS can be diagnosed in utero, most cases have been confirmed officially at autopsy [4,7]. Facial qualities are identifiable at 18 weeks of gestation making diagnosis in the early first trimester difficult [4]. However, micrognathia is evident at 13 weeks of gestation [4,7,10]. Criteria for detecting FFS in the antenatal period have been refined over time. Paladini et al. initially recommended diagnosis with femoral hypoplasia and ≥ 1 major craniofacial abnormality [7]. Johnson et al. updated the current guideline for detection with the following criteria: femoral hypoplasia plus the presence of two or more of the following facial anomalies (upslanting eyes, hypoplastic alae nasi with broad tipped nose, thin upper lip with long philtrum, and small mandible and mouth) [4]. Proper timely diagnosis of this disabling condition is important to give parents time to discuss termination, adoption, or preparation to care for their child after birth. However, taking our married 42-year-old, well-adapted, adult patient into consideration, further reflection may be warranted that patients with FFS can live relatively long, fruitful lives.

The etiology of FFS has not been well defined. However, a strong association with maternal diabetes is proposed with an incidence of 28-38% [2-4,7]. In our case, the patient was born to parents of no familial relationship. The patient’s mother was a 28-year-old heavy smoker with unknown gestational diabetes status at the time of delivery. Oligohydramnios and in utero exposure to radiation, illicit substances, and viral infections have been suggested as other causes for the associated abnormalities seen in this condition, suggesting multifactorial inheritance [1,3,6]. Luisin et al. emphasized the persistent difficulty in distinguishing key features on ultrasound and the need for biological and genetic markers to accurately diagnose FFS [3]. Autosomal recessive inheritance in one family and autosomal dominant inheritance in two families have been described [2,3]. Recent developments have been made in exploring a potential genetic link to FFS. Chromosomal rearrangement involving the deletion or duplication of chromosome 2q37.2 - 2q37.3 was discovered in persons born to non-diabetic mothers [3,4,14]. Specifically, 2q37.2 duplication was noted to be responsible for bilateral femur shortening [3,4,14]. Interestingly, a de novo missense mutation in the *DONSON* gene (Trp2228Leu), which codes for a replication fork protein, was noted in a patient with FFS [11]. Ultimately, more genetic studies are required to elucidate the genetic basis of the multifactorial inheritance of FFS [11].

FFS has variable phenotypic expression with musculoskeletal, craniofacial, and spinal malformations mostly observed. Involvement of other organ systems such as genitourinary, central nervous, and cardiac are also recognized. Gastrointestinal involvement is possible but is only briefly mentioned in three cases. Two instances were of intestinal malrotation, in one instance a 31-week-old male had a medially located appendix, the other was discovered during neonatal autopsy described as a patient born without a rectum or anus in which the blind-ended sigmoid colon was attached to the uterus. However, the upper gastrointestinal tract was noted as normal [12]. A third case reported was of a newborn with characteristic features who later developed GERD [13].

GERD may be more prevalent in patients with FFS than previously reported. It is likely that gastrointestinal manifestations are exacerbated by craniofacial and cervical anomalies. For example, a short neck means a shorter esophagus, which is more prone to constant gastric acid irritation from reflux leading to pathogenic changes and eventual neoplasm.

Chronic GERD results in Barrett’s esophagus (BE), a precursor to esophageal adenocarcinoma (EAC) in which metaplastic columnar epithelium replaces the stratified squamous epithelium normally present in the distal esophagus [15]. BE intestinal metaplasia is the only recognized precursor for the development of EAC [15-16]. Thus, patients with symptoms of GERD should be identified, as this is the most important risk factor for the development of BE, which can evolve to EAC [15]. Obesity, specifically central adiposity, is another key risk factor for the development of EAC due to its contribution to acid reflux and adipocytokines resulting in a proinflammatory state [15]. EAC has a poor prognosis with a 42% survival rate after one year [16] and a five-year survival rate below 20% [16]. Currently, endoscopy screening guidelines are recommended for patients greater than 50 years old [16]. Young patients (less than 50 years old) diagnosed with EAC commonly had current GERD symptoms (55%) and were obese (48%) [16], both of which were present in our young 42-year-old patient. It should also be noted that the patient had a hiatal hernia, which may have likely exacerbated the patient’s GERD. Therefore, it is likely that the patient’s GERD, obesity, and hiatal hernia were the predisposing factors that were worsened by her FFS and led to the development of EAC. FFS impacted her ambulation, predisposing her to obesity, and influenced her severely shortened esophageal anatomy, both factors predisposed her to develop chronic unremitting GERD. Therefore, FFS indirectly caused the development of EAC. 

Concerningly, EAC is usually detected at an advanced stage when symptoms manifest [17]. Early diagnosis is the most effective method of improving survival in patients with EAC [15], such as recognizing patients at high risk for BE and performing a proper endoscopic evaluation to monitor progression in patients displaying BE [15, 18]. Therefore, early detection is principal to delaying progression to EAC. However, guidelines have not been established for the management of EAC in FFS given the incredible rarity of this condition [3]. The longstanding GERD, BE, and diagnosis of advanced malignant EAC at a young age present in this patient highlight the importance of appropriate monitoring and treatment of GERD in patients with FFS.

## Conclusions

FFS is a rare congenital musculoskeletal disorder. In addition to the classic femur and facial aberrations, other skeletal deformities and problems with visceral organ systems are possible. Although FFS is rare in clinical practice, clinical guidelines in pediatric and adulthood should be established for proper FFS evaluation and management over time. As seen in this case, patients with FFS who live into adulthood, may be at increased risk of gastroesophageal reflux disease and later adenocarcinoma. Therefore, in patients with FFS complaining of progressive dysphagia and GERD, a high degree of suspicion is imperative for early detection of esophageal adenocarcinoma. To the best of our knowledge, this is the first documented case of FFS in an adult living to 42 years of age. Further documentation of adult patients with FFS and associated co-morbidities must be reported in order to increase the known literature on FFS, establish guidelines, and ultimately provide better patient care.
